# *Limosilactobacillus reuteri* DSM 17938 supplementation and SARS-CoV-2 specific antibody response in healthy adults: a randomized, triple-blinded, placebo-controlled trial

**DOI:** 10.1080/19490976.2023.2229938

**Published:** 2023-07-04

**Authors:** Richard A. Forsgård, Julia Rode, Karin Lobenius-Palmér, Annalena Kamm, Snehal Patil, Mirriam G. J. Tacken, Marleen A. H. Lentjes, Jakob Axelsson, Gianfranco Grompone, Scott Montgomery, Robert J. Brummer

**Affiliations:** a Nutrition-Gut-Brain Interactions Research Centre, School of Medical Sciences, Faculty of Medicine and Health, Örebro University, Örebro, Sweden; b Clinical Epidemiology and Biostatistics, School of Medical Sciences, Faculty of Medicine and Health, Örebro University, Örebro, Sweden; c Wageningen Bioveterinary Research, Wageningen University and Research, Lelystad, The Netherlands; d BioGaia AB, Stockholm and Lund, Sweden; e Clinical Epidemiology Division, Department of Medicine, Solna, Karolinska Institutet, Stockholm, Sweden; f Department of Epidemiology and Public Health, University College London, London, UK

**Keywords:** Probiotics, SARS-CoV-2, COVID-19, immunology, antibody

## Abstract

Studies have shown that probiotics can decrease the symptoms of respiratory tract infections as well as increase antibody responses following certain vaccinations. We examined the effect of probiotic supplementation on anti-SARS-CoV-2 specific antibody responses upon SARS-CoV-2 infection as well as after COVID-19 vaccination. In this randomized, triple-blinded, placebo-controlled intervention study with a parallel design, 159 healthy adults without prior SARS-CoV-2 infection or COVID-19 vaccination and any known risk factors for severe COVID-19 were randomly allocated into two study arms. The active treatment arm consumed a probiotic product containing a minimum of 1 × 10^8^ colony-forming units of *Limosilactobacillus reuteri* DSM 17938 + 10 μg vitamin D3 twice daily for 6 months. The placebo arm consumed identical tablets containing only 10 μg vitamin D3. Anti-SARS-CoV-2 specific antibodies and virus neutralizing antibody titers were analyzed from blood samples collected at baseline, after 3 months, and after 6 months. Differences in serum antibody titers between the two study arms were tested with independent t-test using log-transformed values. In the intention-to-treat (ITT) analysis, SARS-CoV-2 infected individuals in the active treatment arm (*n* = 6) tended to have higher serum anti-spike IgG (609 [168–1480] BAU/ml vs 111 [36.1–1210] BAU/ml, *p* = 0.080) and anti-receptor binding domain (RBD) IgG (928 [212–3449] BAU/ml vs (83.7 [22.8–2094] BAU/ml, *p* = 0.066) levels than individuals in the placebo arm (*n* = 6). Considering individuals who were fully vaccinated with mRNA-based COVID-19 vaccines, the active treatment arm (*n* = 10) exhibited significantly higher serum levels of anti-RBD IgA (135 [32.9–976] BAU/ml vs 61.3 [26.7–97.1] BAU/ml, *p* = 0.036) than the placebo arm (*n* = 7) >28 days postvaccination. Supplementation with specific probiotics might improve the long-term efficacy of mRNA-based COVID-19 vaccines via enhanced IgA response.

## Introduction

The global outbreak of the coronavirus disease 2019 (COVID-19) caused by the novel severe acute respiratory syndrome coronavirus 2 (SARS-CoV-2) presents a serious threat to public health with at least 6.9 million people dying of the disease.^1^ Despite the emergence of antiviral therapies and the advances made in treating patients with acute COVID-19, the best strategy to ease disease burden both on an individual and on a societal level is a high level of population immunity through mass vaccination campaigns. The widely used messenger-RNA (mRNA)-based COVID-19 vaccines, namely Pfizer-BioNTech’s BNT162b2 and Moderna’s mRNA-1273, elicit a strong cellular and humoral immune response which significantly decreases the risk for hospitalization and death.^2–5^ However, the vaccine-induced humoral response wanes over time with numerous studies showing significantly decreased blood anti-SARS-CoV-2 immunoglobulin (Ig) levels 6–8 months after vaccination.^6^ Considering that mounting evidence suggests that low levels of anti-SARS-CoV-2 antibodies are associated with increased susceptibility to reinfections and breakthrough infections,^7–9^ measures to boost and prolong antibody titers could provide substantial population-level benefits in the disease management of COVID-19.

Probiotics are live microorganisms which “when administered in adequate amounts can confer health benefits to the host”.^10^ Clinical studies in different populations have reported that probiotic supplementation can reduce the occurrence and symptoms of viral respiratory tract infections possibly via mechanisms involving probiotic-mediated changes in innate immunity.^11^ Additionally, clinical studies in healthy humans have demonstrated that specific probiotics can affect humoral immune responses after certain vaccinations.^12,13^ For example, several studies have reported significantly increased serum levels of influenza-specific antibody titers and higher seroconversion rates following probiotic supplementation.^13–18^ As summarized in a recent review article,^13^ these results have raised interest in probiotics as a potential adjuvant to improve vaccine responses, especially in elderly people. However, due to heterogeneity in study designs (probiotic strain, duration of the intervention, type of vaccine) and inconsistency in observed effects, the effectiveness of probiotics as vaccine adjuvants seems to vary depending on the type of vaccine and the specific disease.

The primary aim of this study was to investigate whether supplementation with *Limosilactobacillus reuteri* (*L. reuteri*) DSM 17938 associated with 10 μg vitamin D3 increases the anti-SARS-CoV-2 antibody response upon infection. However, because the study period (December 2020-September 2021) overlapped with the mass vaccination campaign against COVID-19 in Sweden, this allowed us to also examine vaccine-induced antibody responses.

## Results

### Participant characteristics

**Figure 1.**
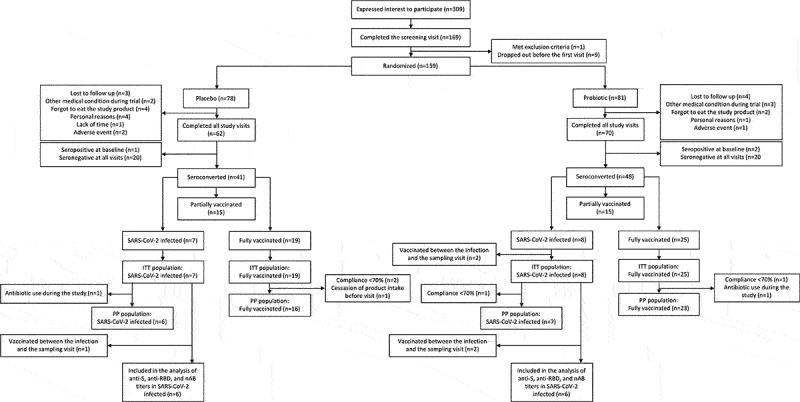
Flowchart showing the number of study participants from the start of the trial to the analyzed populations. ITT=Intention-to-treat, PP=Per-protocol.

A total of 159 participants were enrolled in the trial and 132 of these (108 females, 24 males) completed all three study visits. Of the 27 dropouts, three dropped out because of adverse events, the rest either informed us of their withdrawal or were lost during follow-up (Figure 1). The participant characteristics of the ITT and per-protocol populations in each group are listed in Table 1.

**Table 1.** Participant characteristics.

		ITT population		PP population	
Group		Placebo	Probiotic	Placebo	Probiotic
n		41	48	34	43
Age (years)		48 (29–60)	51.5 (21–60)	49 (29–60)	52 (21–60)
Sex	Female	33	41	29	38
	Male	8	7	5	5
BMI	Underweight (<18.5)	-	-	-	-
	Normal (18.5–24.9)	23	28	19	25
	Overweight (25–29.9)	14	18	11	16
	Obese (>30)	4	2	4	2
Subgroup	Infected	7	8	6	7
	1 vaccine dose	15	15	12	13
	2 vaccine doses	19	25	16	23
Compliance (%)		90 (57–113)	90 (59–100)	92 (72–106)	91 (71–100)
Total daily energy intake (mJ)		7.6 (4.7–11.8)	8.5 (4.6–16.2)	7.7 (4.7–11.8)	8.4 (4.6–14.2)
Daily intake (% of total daily energy intake)	Carbohydrates	43.1 (20.6–56.8)	42.1 (24.9–60.4)	43.0 (20.6–52.7)	41.6 (24.9–52.5)
	Protein	16.8 (11.0–20.9)	16.5 (11.1–20.9)	16.8 (13.5–20.9)	16.5 (11.1–20.9)
	Fat	35.0 (26.6–55.4)	36.9 (21.5–51.0)	35.3 (28.2–55.4)	37.3 (30.2–47.7)
	Fiber	2.7 (1.8–5.1)	2.6 (1.4–4.5)	2.7 (1.8–5.1)	2.6 (1.4–4.1)
	Alcohol	1.4 (0–10.3)	1.5 (0–7.7)	1.3 (0–10.3)	1.5 (0–7.7)

BMI = body mass index, ITT = intention-to-treat, PP = per-protocol.

### Serum anti-SARS-CoV-2 specific antibody titers after infection

The study participants self-reported 14 PCR-confirmed SARS-CoV-2 infections and one additional infection was confirmed by the emergence of anti-N IgG antibodies (the subject was symptomatic but had tested negative). However, three subjects (two in the probiotic group, one in the placebo group) were vaccinated between their infection and sampling which meant that their anti-S IgG/IgA, anti-RBD IgG/IgA, and virus neutralizing antibody (nAB) results were excluded from these analyses leaving six participants in each study arm for these analyses.

**Figure 2.**
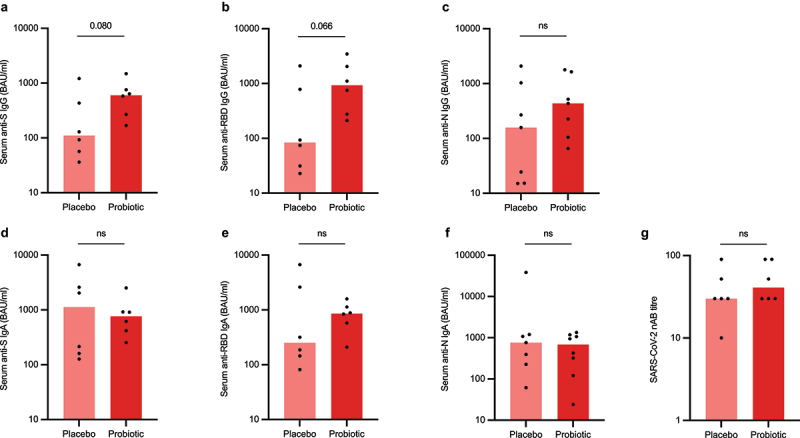
Serum anti-SARS-CoV-2 specific antibody (a-f) and virus-neutralizing antibody levels (g) in study participants who were infected during the study period (intention-to-treat population). S = spike, RBD = receptor-binding domain, *N* = nucleocapsin, nAB = neutralizing antibody. Statistical comparisons between the groups were performed with independent t-test using log-transformed values except nAB titers which were compared with non-parametric Mann-Whitney test. The bar plots show median, and the dots represent individual values within the group.

The median serum anti-S IgG titer was 609 (168–1,480) BAU/ml in the active treatment arm (*n* = 6) which was higher than in the placebo arm (111 [36.1–1,210] BAU/ml, *n* = 6) but this difference did not reach statistical significance (*p* = 0.080) (Figure 2a). Similarly, the active treatment arm exhibited higher levels of serum anti-RBD IgG (928 [212–3,449] BAU/ml, *n* = 6) than the placebo arm (83.7 [22.8–2,094] BAU/ml, *n* = 6) but again, this difference did not reach statistical significance (*p* = 0.066) (Figure 2b). The median serum anti-N IgG concentration was 331 (25.2–1,784) BAU/ml in the active treatment arm (*n* = 8) and 158 (15.1–2,086) BAU/ml in the placebo arm (*n* = 7, *p* = 0.253) (Figure 2c). There were also no statistically significant differences in serum virus-specific IgA antibodies nor nAB titers between the study arms (Figure 2d–g). All measured antibody titers for the two study arms and corresponding p-values in the ITT and per-protocol population are presented in Supplementary Table S1.

### Symptom duration and severity after SARS-CoV-2 infection

All infected participants in both study arms experienced only ambulatory disease symptoms with median symptom duration being 14 (12–26) days in the placebo arm and 17 (6–31) days in the probiotic group, but there was no statistically significant difference between the groups. The most common individual symptom was fatigue which was reported by all infected participants in both study arms. The frequencies of individual symptoms are presented in Supplementary Table S1.

### COVID-19 vaccinations during the study period

Of the 132 participants who completed all the study visits, 95 received at least one vaccine dose during the study period. Most participants received the Pfizer-BioNTech’s BNT162b2 vaccine (*n* = 84/95) with the rest receiving either Moderna’s mRNA-1273 vaccine (*n* = 7) or Astra-Zeneca’s AZD1222 vaccine (*n* = 3) or a combination of AZD1222 and BNT162b2 (*n* = 1). Of the 95 vaccinated individuals, 49 received two vaccine doses during the study period. A total of five of the fully vaccinated participants were also infected prior to vaccination and thus their antibody values were excluded from this analysis, leaving a total of 44 fully vaccinated individuals in this subgroup.

### Serum anti-SARS-CoV-2 specific antibody titers after full vaccination

**Figure 3.**
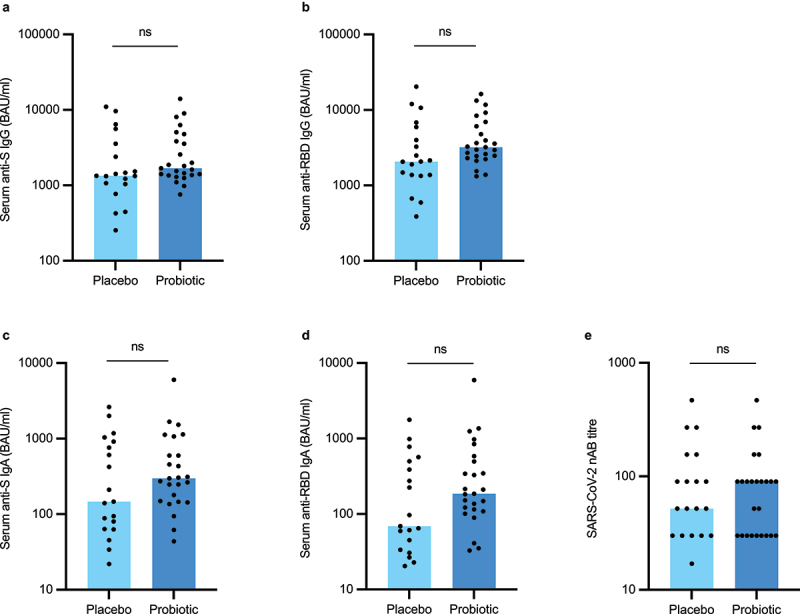
Serum anti-SARS-CoV-2 specific antibody (a-d) and virus-neutralizing antibody levels (e) in study participants who received two vaccine doses during the study period (intention-to-treat population). S = spike, RBD = receptor-binding domain, nAB = neutralizing antibody. Statistical comparisons between the groups were performed with independent t-test using log-transformed values except nAB titers which were compared with non-parametric Mann-Whitney test. The bar plots show median, and the dots represent individual values within the group.

In the fully vaccinated, the median serum anti-S IgG titer was 1,680 (756–14,000) BAU/ml in the active treatment arm (*n* = 25) and 1,340 (253–11,000) BAU/ml in the placebo arm (*n* = 19) (*p* = 0.221) (Figure 3a). The median serum anti-RBD IgG levels were 3,215 (1,321–16,258) BAU/ml and 2,068 (387–20,341) BAU/ml in the active treatment and the placebo arm, respectively (*p* = 0.127) (Figure 3b). The median anti-S IgA was 297 (43.9–5,996) BAU/ml in the active treatment arm and 147 (21.9–2,617) in the placebo arm (*p* = 0.266) (Figure 3c). There were also no statistically significant differences between the study arms in serum levels of anti-RBD IgA (*p* = 0.118) (Figure 3d). Similarly, we did not observe a statistically significant difference in the nAB titers between the groups (Figure 3e). All measured antibody titers for the two study arms and corresponding p-values in the ITT and per-protocol population are presented in Supplementary Table S2.

**Figure 4.**
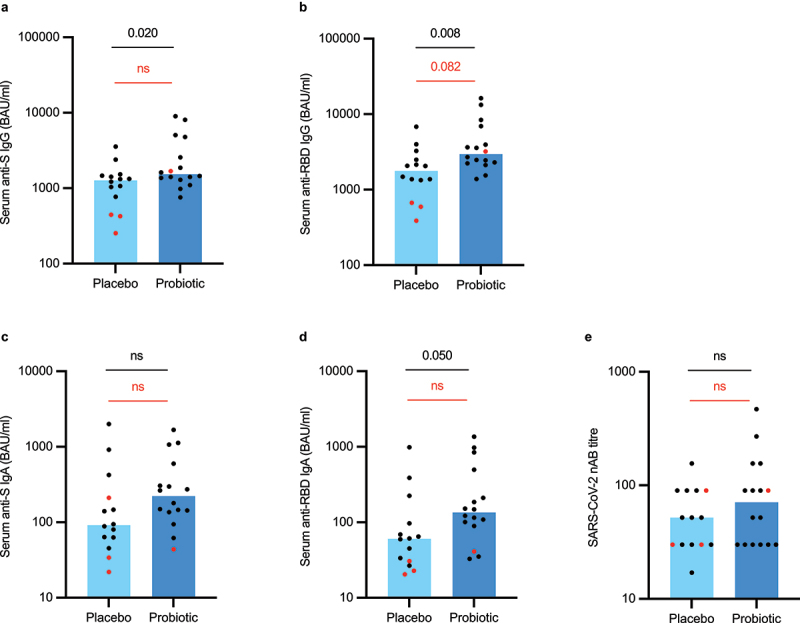
Serum anti-SARS-CoV-2 specific antibody (a-d) and virus-neutralizing antibody (e) levels in study participants who received two vaccine doses during the study period and were sampled more than 14 days after the second dose (intention-to-treat population). The red dots represent individuals who were either partly or fully vaccinated with the Astra-Zeneca’s AZD1222 vaccine. S = spike, RBD = receptor-binding domain, nAB = neutralizing antibody. Statistical comparisons between the groups were performed with independent t-test using log-transformed values except nAB titers which were compared with non-parametric Mann-Whitney test. Black p-values refer to the whole population and red p-values to the population who received only Pfizer-BioNTech’s BNT162b2 vaccine or Moderna’s mRNA-1273 vaccine. The bar plots show median, and the dots represent individual values within the group.

**Figure 5.**
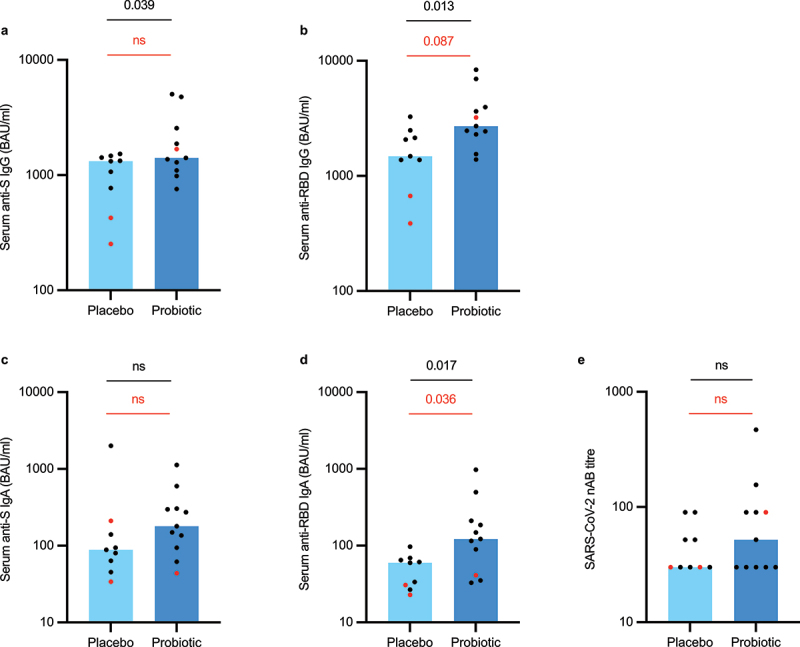
Serum anti-SARS-CoV-2 specific (a-d) and virus-neutralizing (e) antibody levels in study participants who received two vaccine doses during the study period and were sampled more than 28 days after the second dose (intention-to-treat population). The red dots represent individuals who were either partly or fully vaccinated with the Astra-Zeneca’s AZD1222 vaccine. S = spike, RBD = receptor-binding domain, nAB = neutralizing antibody. Statistical comparisons between the groups were performed with independent t-test using log-transformed values except nAB titers which were compared with non-parametric Mann-Whitney test. Black p-values refer to the whole population and red p-values to the population who received only Pfizer-BioNTech’s BNT162b2 vaccine or Moderna’s mRNA-1273 vaccine. The bar plots show median, and the dots represent individual values within the group.

Based on the assumption that age, BMI, time from vaccination to sampling, and time between the two vaccine doses can influence the measured antibody titers, we performed a multivariate linear regression analysis to study the associations between these variables. This analysis revealed that only time from vaccination had a statistically significant effect on all measured virus-specific antibody class levels except nABs. Thus, we decided to split the data according to the time elapsed since vaccination. First, we investigated individuals in the fully vaccinated subgroup who had received their second vaccine dose more than 14 days before blood sampling which is generally considered as the time regarded for full vaccination effect. This analysis showed that the active treatment arm (*n* = 16) had significantly higher serum levels of anti-S IgG (*p* = 0.02), anti-RBD IgG (*p* = 0.008), and anti-RBD IgA (*p* = 0.050) than the placebo arm (*n* = 14) (Figure 4). However, these differences appeared to arise largely due to three individuals with low antibody titers in the placebo arm who were fully vaccinated with the AZD1222 vaccine. After reanalysis including only individuals who were fully vaccinated with the mRNA-based vaccines BNT162b2 or mRNA-1273, we observed a non-significant trend (*p* = 0.082) for higher anti-RBD IgG levels in the active treatment arm (*n* = 15) compared to the placebo arm (*n* = 11). Next, to investigate further the long-term effects of probiotic supplementation on vaccine-induced antibody responses, we split the data again to only include fully vaccinated individuals who had received their second vaccine dose more than four weeks (28 days) before blood sampling (Figure 5). The active treatment arm exhibited significantly higher serum anti-S IgG (*p* = 0.039), anti-RBD IgG (*p* = 0.013), and anti-RBD IgA (*p* = 0.017) levels than the placebo arm. Again, the statistical differences between the groups were mostly driven by the relatively low antibody levels observed in individuals who were fully vaccinated with the AZD1222 vaccine. When only including the recipients of two mRNA vaccine doses, the probiotic arm (*n* = 10) still showed significantly higher anti-RBD IgA levels than the placebo group (*n* = 7, *p* = 0.036).

### Correlations between anti-SARS-CoV-2-specific antibody titers and time from vaccination

All measured virus-specific antibody levels showed a significant negative correlation with the number of days elapsed between full vaccination and blood sampling (Supplementary Figure S1). In addition, the serum levels of anti-S IgG, anti-RBD IgG, anti-S IgA, and anti-RBD IgA exhibited a significant positive correlation with the measured nAB titers (Supplementary Figure S2).

## Discussion

The aim of this randomized, triple-blinded, placebo-controlled study was to explore whether probiotic supplementation could improve anti-SARS-CoV-2 antibody response upon infection in healthy adults. To our knowledge, this is the first study to analyze the effects of probiotic supplementation on different anti-SARS-CoV-2 antibody classes, including virus-neutralizing antibodies, throughout the whole seroconversion event.

Overall, we observed a total of 15 SARS-CoV-2 infections during the study period and were able to analyze infection-induced anti-S and anti-RBD IgG levels in 12 subjects. This analysis showed a non-significant trend for higher serum anti-S (*p* = 0.080) and anti-RBD (*p* = 0.066) IgG titers in the active treatment arm compared to the placebo arm. Unfortunately, these data have inadequate statistical power to identify unequivocal differences between the groups, but the observations are in line with the findings of Gutiérrez-Castrellón et al.^19^, the only other study to our knowledge to report the effects of probiotics on SARS-CoV-2-specific antibody response following COVID-19. In their randomized, quadruple-blinded clinical trial including 300 symptomatic COVID-19 outpatients who started taking a probiotic mixture or a placebo mixture after testing positive for SARS-CoV-2, the probiotic group exhibited higher levels of anti-S IgG and IgM than the placebo group after 15 and 30 days.^19^ However, in contrast to our findings, the authors also reported significant improvements in clinical outcomes, such as symptom duration, viral load, and lung infiltrates, in the probiotic group whereas we did not observe any group-level differences in symptom duration or severity. This discrepancy arises most likely due to the low number of infections in our cohort which did not allow a sufficiently powered investigation into the clinical features of these infections. Additionally, because our study population consisted of relatively young individuals without any comorbidities for severe COVID-19, the overall symptom burden in our study was small which possibly further masked any potential differences between the groups. Nevertheless, our results seem to support the growing pre-clinical and clinical evidence of probiotics’ ability to enhance humoral immunity albeit the clinical significance of this finding remains uncertain.

Although this study was initially designed to investigate virus-specific antibody responses following SARS-CoV-2 infection, the start of the mass vaccination campaign against COVID-19 in the spring of 2021 in Sweden provided the opportunity to analyze vaccine-induced antibody responses. Reflecting Sweden’s COVID-19 vaccination policies, most participants in our cohort were vaccinated during the intervention period with the mRNA-based COVID-19 vaccines, BNT162b2 and mRNA-1273, which lead to the production of IgA and IgG against the spike antigens (S and RBD) of SARS-CoV-2.^20–22^ To our knowledge, only two other randomized, double-blinded, placebo-controlled studies have previously examined the effects of probiotic supplementation on spike-specific antibody responses after COVID-19 vaccination.^23,24^ In a recently published study on Spanish health-care workers, individuals who consumed *Loigolactobacillus coryniformis* K8 had significantly higher serum levels of anti-S IgG levels ≥81 days post-first vaccine dose compared to individuals in the placebo arm.^23^ However, this sub-group analysis also included several individuals who had started in the trial between their first and second vaccine doses, and since the time-dependent comparisons were made relative only to the first vaccine dose, it is unclear how the probiotic affected the waning of antibody titers after full vaccination.^23^ In another study using the same probiotic strain, Fernández-Ferreiro et al. analyzed serum levels of anti-RBD IgG and IgA in 200 nursing home residents (>60 years old) who started taking *Loigolactobacillus coryniformis* K8 or a placebo product between the two doses of the BNT162b2 COVID-19 vaccine.^24^ They observed significantly higher anti-RBD IgG levels in individuals in the probiotic group who were infected after their first vaccine dose but no differences in vaccine-induced antibody levels other than a non-significant trend (*p* = 0.082) for higher anti-RBD IgA titers in a subgroup analysis of fully vaccinated > 85-year-olds in the probiotic group.^24^ Due to the low number of cases, we could not make any group-level comparisons regarding the combined effects of natural infection and vaccination on antibody levels. It should be noted though that those infected individuals who later received one vaccine dose exhibited similar anti-S IgG titers as individuals who were uninfected but fully vaccinated individuals, whereas natural infection with full vaccination was associated with significantly higher anti-S IgG levels than two vaccine doses alone (data not shown).

In our initial statistical analysis, we did not observe any differences in vaccine-induced anti-S and anti-RBD IgG or IgA responses in fully vaccinated individuals between the study arms. Also, we did not measure any group-level differences in SARS-CoV-2 nAB titers after full vaccination. It is important to note that although the virus-specific IgG and IgA levels correlated positively with the measured nAB titers (Supplementary Information), virus neutralization was determined against the B.1.617.2 (Delta) variant that has shown reduced sensitivity to vaccine generated antibodies.^25^ In addition, due to our study design, the sampling visits were not fixed relative to any vaccination events. This might have introduced bias into the initial analysis considering that the vaccine-induced antibody response seems to peak around 21–28 days after the second dose after which postvaccination antibody levels progressively wane.^6^ Thus, we decided to analyze group-wise differences at two time points: after 14 days post-second dose (WHO’s cutoff for full vaccination) and after 28 days (peak/post-peak antibody response). These analyses showed significantly elevated serum anti-S IgG as well as anti-RBD IgG and IgA levels in the probiotic arm compared to the placebo arm. However, the placebo arm included three individuals who were fully vaccinated with the AZD1222 vaccine that elicits a weaker humoral response against the viral spike antigens than the mRNA COVID-19 vaccines.^26^ After removing these individuals and one participant from the probiotic arm who received a mixed prime-boost schedule of AZD1222 and BNT162b2 from the analyses and subsequently analyzing only those participants who received two doses of mRNA-based vaccines, the probiotic group still exhibited significantly higher serum anti-RBD IgA titers than the placebo group when more than 28 days had passed from the second vaccine dose. Additionally, we observed a non-significant trend for higher anti-RBD IgG levels in the probiotic arm in this cohort as well as in the post-14 days cohort. Although we could not measure any significant differences in nAB titers between the study arms in these cohorts, higher anti-RBD antibodies potentially have important clinical significance for long-term vaccine efficacy considering their role for preventing breakthrough infections. In a recent study, Sheikh-Mohamed et al. reported that fully vaccinated individuals who experienced a breakthrough infection had significantly lower anti-S/RBD IgA titers at 2–4 weeks after the second vaccine dose than uninfected controls.^22^ In their cohort, the median IgA level before the infection was 153 BAU/ml for anti-S IgA and 162 BAU/ml for anti-RBD IgA in the breakthrough infection group whereas in the uninfected group the median levels of anti-S and anti-RBD IgA reached 417 BAU/ml and 495 BAU/ml, respectively.^22^ Interestingly, when compared to our values, in the probiotic group 60% of the fully vaccinated participants (9/15) exhibited anti-S IgA titers > 153 BAU/ml 20–69 days post-dose 2 compared to only 27% (3/11) in the placebo group. Similarly, 40% of the fully vaccinated participants in the probiotic group (6/15) had higher anti-RBD IgA levels than 162 BAU/ml after the second vaccine dose compared to 27% (3/11) of the participants in the placebo group. Overall, these findings would suggest that *L. reuteri* DSM 17938 supplementation might increase the long-term efficacy of COVID-19 vaccines against breakthrough infections via enhanced IgA response after vaccination. This potentially could have considerable benefits for at-risk individuals and for the prevention of community outbreaks. However, as our study was not designed to investigate this question, this hypothesis should be tested in a larger cohort with fixed sampling times and a controlled postvaccination observational period.

To the best of our knowledge this the first study to investigate the effects of probiotic supplementation on anti-SARS-CoV-2 antibody response, including virus-neutralizing antibody titers, in exclusively SARS-CoV-2 antigen-naïve individuals. Nevertheless, this study has several strengths and limitations that should be considered when interpreting the results. Regarding the strengths, in the previously mentioned studies, the intervention period commenced after a positive test result^19^ or the first vaccine dose^23,24^ whereas in our study all participants started the trial prior to infection or vaccination. Also, we were able to show probiotic-induced benefits in vaccine responses in a study cohort consisting of relatively young (<60) and healthy adults increasing the generalization of these results to the overall population. However, this could also be seen as a limitation. Also, as we did not examine the participants’ fecal microbiota composition, we cannot make any associations between the resident gut microbes and immune responses. Although these analyses might have been interesting, considering that probiotic supplementation does not appear to affect the fecal microbiota composition^27^ as well as the importance of small intestinal microbiota-host interaction for immune responses, it is uncertain whether fecal microbiota analysis would have provided additional strength to our findings. Regarding other limitations, this study was not initially designed to examine vaccine-induced antibody responses which led to differences in vaccine types and vaccine dose intervals. However, we did not observe any significant differences between the groups in vaccine dose intervals, mainly because most participants followed had exactly 42 days between vaccine doses as recommended by the Swedish health authorities. Secondly, as we initially aimed to observe and analyze SARS-CoV-2 infections during the study period, the sampling visits occurred at 3 and 6 months after starting the intervention period meaning that they occurred randomly relative to the participants’ dates of vaccination. Due to the time sensitivity of the antibody response, the participants might have been at different stages of seroconversion when coming for the sampling visit. Although we did not observe any significant differences in vaccination-to-sampling times between the groups, it is likely that some participants had not reached peak antibody levels following vaccination. Overall, these limitations in study design likely decreased our statistical power and led to subgroup analyses with rather small number of participants necessitating caution when interpreting the results.

In conclusion, supplementation with a probiotic strain *L. reuteri* DSM 17938 was associated with statistically non-significant increase in serum anti-S and anti-RBD IgG titers upon infection with SARS-CoV-2. In addition, in our subgroup analysis of fully vaccinated individuals with more than 28 days postvaccination, the individuals who consumed *L. reuteri* DSM 17938 exhibited statistically significantly higher anti-RBD IgA levels than individuals who consumed the placebo product. Together, these results suggests that specific probiotic supplementation might enhance long-term immune responses, particularly to mRNA-based COVID-19 vaccines, but these findings should be validated in larger studies with fixed sampling times postvaccination.

## Materials and methods

### Study design

The study was a single-center, randomized, triple-blinded, placebo-controlled intervention study with a parallel design and two study arms. The primary outcome parameter was the effect of probiotic supplementation on serum anti-spike (S) IgG levels following SARS-CoV-2 infection. The secondary outcomes included the serum levels of other anti-SARS-CoV-2 specific antibodies as well as the severity and duration of COVID-19 symptoms. COVID-19 vaccine-induced antibody responses were included as exploratory outcome measures.

**Figure 6.**
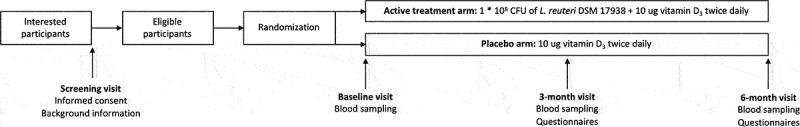
Study design.

After giving their informed consent and completing the screening procedures, participants eligible for the study were randomized into the two study arms (placebo, active treatment) prior to undergoing a baseline visit before the start of the intervention period (Figure 6). During the 6-month intervention period, the participants attended study visits at 3 months into the intervention as well as at the end of intervention (at 6 months). Blood samples were collected at each visit. In addition, during the whole intervention period, the participants were asked to fill out a weekly questionnaire in which they were asked to record any possible symptoms of COVID-19 and symptom severity as well as whether they have received any vaccinations for COVID-19. The study protocol was registered at clinicaltrials.gov (NCT04734886).

### Subjects

Participants were recruited via advertisements at Örebro University Hospital and Örebro University campus as well as via social media. All participants signed a dated informed consent prior to any study-related activities. The participants’ eligibility for the study was assessed via questionnaire. The study participants were instructed to avoid any changes to their habitual diet and lifestyle during the study. Intake of probiotics or nutritional supplements containing pro- and/or prebiotics was not allowed from 4 weeks before the screening. The inclusion and exclusion criteria are listed in Table 2.

**Table 2.** Inclusion and exclusion criteria for the study.

Inclusion criteria	Exclusion criteria
Signed informed consent	Previous diagnosis of COVID-19 (by positive PCR) or previous confirmation of seropositivity to SARS-CoV-2
Age 18–60 years	Body Mass Index over 35 or under 16
	Current diagnosis of cancer or ongoing cancer treatment in the last 12 months
	Diabetes mellitus
	Cardiovascular disorder in need of pharmaceutical treatment
	Chronic kidney disease
	Chronic lung disease with decreased lung capacity
	Chronic liver disease with liver cirrhosis
	Current diagnosis of dementia, severe depression, major psychiatric disorder, or other incapacity for adequate cooperation
	Chronic neurological/neurodegenerative disease (e.g., Parkinson’s disease)
	Decreased function of the adrenal cortex (e.g., Addison’s disease)
	Autoimmune disease (e.g., rheumatoid arthritis)
	Chronic pain syndromes (e.g., fibromyalgia)
	Pregnancy or breast-feeding
	Immunodeficiency due to disease or ongoing medical treatment
	Regular intake of anti-inflammatory and/or other immunosuppressive medication within the last 3 months
	Use of anti-depressants within the last 3 months
	Antimicrobial treatment within the last 12 weeks before baseline sampling
	Regular intake of probiotics, as well as nutritional supplements or herb products that might affect intestinal function within the last 4 weeks if the investigator considers that those could affect study outcome
	Inability to maintain current diet and lifestyle during the study period
	Alcohol or drug abuse

### Study intervention

The active treatment arm consumed a probiotic tablet containing a minimum of 1 × 10^8^ colony-forming units (CFU) of *L. reuteri* DSM 17938 + 10 μg vitamin D3 (L. *reuteri* Protectis®, BioGaia, Lund, Sweden) twice daily. The placebo arm consumed tablets of identical content except for the absence of the probiotic strain. Compliance was assessed by asking the participants to return any unused product at the end of the study. When the participants failed to do so, they were instructed to count the remaining tablets at home and report the number of tablets left to the study team.

### Randomization and masking

Subjects were randomized to the two study arms in the order of their enrollment in the study using a computerized randomization list with a random block size of four. The study products were packed into sequentially numbered containers labeled by a BioGaia AB employee not involved in the study. This person also created and controlled the randomization key which was not revealed until all laboratory analyses were completed. Allocation was revealed for statistical analysis. The investigators and study participants were blinded to the group identities throughout the study.

### Blood sampling

Blood samples were withdrawn into Vacuette CAT serum separator clot activator tubes (Greiner Bio-One, Austria), centrifuged, and the separated serum was aliquoted and stored at −80°C until analysis.

### Analysis of serum anti-SARS-CoV-2 antibody titers

The serum IgG titers against the S epitope of SARS-CoV-2 were analyzed with Liaison XL (Diasorin, Italy) at Örebro University Hospital according to the manufacturer’s instructions.

The serum IgG titers against the nucleocapsin (N) and the spike receptor-binding domain (RBD) epitopes of SARS-CoV-2 were analyzed with MSD V-Plex SARS-CoV-2 Panel 2 (IgG) according to the manufacturer’s instructions.

The serum titers of anti-S, anti-N, and anti-RBD IgA were analyzed with MSD V-Plex SARS-Cov-2 Panel 2 (IgA) according to the manufacturer’s instructions.

### Analysis of SARS-CoV-2 neutralizing antibodies

Serum titers of virus-neutralizing antibodies (nABs) were analyzed with a live virus neutralization test (VNT) which is based on the inhibition of virus growth *in vitro*. The neutralization titer of each sample was determined as the highest average serial dilution able to inhibit > 50% virus growth. The assay details are available in the Supplementary Information.

### Study questionnaires

Individual COVID-19 symptoms’ duration and severity were assessed during the study by a specialized questionnaire where the participants were asked to mark weekly whether they had experienced any possible symptoms of COVID-19. The symptom list included: cough, sore throat, nasal congestion, breathing difficulties, fever, muscle or body ache, loss of taste or smell, diarrhea, nausea, headache, and fatigue. If the participants answered “Yes” to any of those symptoms, they were asked to also fill in when the symptoms started and ended. The questionnaire also included questions if the participant had been tested for COVID-19 during the week (free PCR testing was available to all residents via public health care), if they had visited a health center because of their symptoms, and if they had taken any medications for their symptoms. The participants were also asked to record the time and vaccine type if they had received any against COVID-19 during the week. Symptom severity was assessed according to World Health Organization’s Ordinal Scale for Clinical Improvement where the severity of symptoms is graded on a scale of 0–8. The grade 0 refers to no clinical or virological signs of infection, grades 1–2 to ambulatory disease symptoms, grades 3–4 to mild disease requiring medical treatment at a hospital, grades 5–7 to severe disease, and grade 8 to death from the disease.

At the start of the study, participants were asked to complete a food frequency questionnaire (FFQ) specially designed for the eating habits of the Swedish population. The recall time was the past six months. The FFQ was semi-quantitative (i.e., included questions on portion sizes). Frequency categories varied from never to three or more times daily. In total 183 food items were listed, though certain items were conditional on previous answers given. Completion time was 30–40 minutes. Nutrient intake was calculated using the Swedish food composition table maintained by the Swedish Food Agency. Daily intake of carbohydrates, protein, fat, fiber, and alcohol were converted to their energy equivalent and results are presented as percent of mean daily energy intake (E%).

### Sample size calculation

Based on previous infection rates in the area, we estimated that we would need to recruit 400 participants (200 per arm) to have a minimum of 33 infected participants per arm to demonstrate statistical significance in anti-SARS-CoV-2 antibody levels with 80% power and 95% confidence intervals.

### Safety and adverse events

Both the probiotic and the placebo product were well tolerated: only three participants withdrew from the study because of gastrointestinal complaints after starting in the trial. A total of two of these were in the placebo group, and one in the probiotic group.

### Statistical analyses

To study the effects of probiotic supplementation on SARS-CoV-2 specific antibody response after infection or vaccination, only participants who seroconverted during the study were included in the statistical analyses. Seroconversion was determined by having serum anti-S IgG concentrations above 34 BAU/ml (Örebro University Hospital’s cutoff). Participants who were seropositive at baseline were excluded from the analyses. Participants with serum anti-N IgG titers above 11.8 BAU/ml (manufacturer’s cutoff) in addition to anti-S IgG concentrations > 34 BAU/ml were considered infected. For statistical analyses, participants were divided into three separate sub-groups based on their antibody status and information regarding vaccinations: Infected (serum anti-N IgG > 11.8 BAU/ml, and no reported vaccinations before the visit), partially vaccinated (serum anti-N IgG < 11.8 BAU/ml, serum anti-S IgG > 34 BAU/ml, and reported to have received one vaccine dose before the visit), and fully vaccinated (serum anti-N IgG < 11.8 BAU/ml, serum anti-S IgG > 34 BAU/ml, and reported to have received two vaccine doses before the visit). Antibody values from the study visit in which the participant first fulfilled one of these categories were used in all analyses. To test for group-wise differences in virus-specific antibody levels, the individual antibody values were tested for normality using the D’Agostino-Pearson normality test. If the antibody values failed the normality test, the values were log-transformed and tested again for normality. Based on the results of the normality tests, differences in serum antibody titers between the two study arms were tested with independent t-test using the log-transformed values except the nAB titers which were analyzed with Mann-Whitney test using the raw values. Multivariate linear regression analysis was performed to adjust for age, body mass index (BMI), time between vaccination and sampling, as well as time between the vaccine doses. Correlation between serum IgG and IgA antibody levels and the number of days between sampling and vaccination was analyzed with Pearson’s correlation test. Correlations between nAB titers and IgG and IgA antibody levels, as well as nAB titers and the number of days between sampling and vaccination were analyzed using Spearman’s correlation test. Intention-to-treat (ITT) population was used as the primary analysis. For the per-protocol analysis, participants with major protocol deviations during the study period were excluded. Major protocol deviations were compliance below 70% throughout the trial or running out of study product before the final visit, continuous and prolonged use of anti-inflammatory medication not related to infection symptoms during the study period, continuous and prolonged use of other probiotic or prebiotic supplements during the study period, use of antibiotics during the study period, or any other possible deviations the principal investigator considered as a major breach of the study protocol. All values are reported as median (minimum-maximum) unless otherwise stated. All statistical analyses were performed using GraphPad Prism 9 and group-wise differences were deemed significant if *p* < .05.

## Supplementary Material

Supplemental Material

## Data Availability

Due to the nature of this research, participants of this study did not provide a written consent for their data to be shared publicly, so supporting data is not publicly available.
