# Supplementary material for: Limosilactobacillus reuteri DSM 17938 supplementation and SARS-CoV-2 specific antibody response in healthy adults: a randomized, triple-blinded, placebo-controlled trial
Source: Gut Microbes. 2023 Jul 4;15(1):2229938. doi: 10.1080/19490976.2023.2229938 (PMC10321188; doi:10.1080/19490976.2023.2229938)
Supplement: Supplemental Material [file KGMI_A_2229938_SM9932.docx]

**Supplementary material**

***Limosilactobacillus reuteri* DSM 17938 supplementation and SARS-CoV-2 specific antibody response in healthy adults: a randomized, triple-blinded, placebo-controlled trial**

Richard A Forsgård^1^, Julia Rode^1^, Karin Lobenius-Palmér^1^, Annalena Kamm^1^, Snehal Patil^2^, Mirriam GJ Tacken^3^, Marleen AH Lentjes^1,2^, Jakob Axelsson^4^, Gianfranco Grompone^4^, Scott Montgomery^1,2,5,6^, Robert J Brummer^1^

^1^Nutrition-Gut-Brain Interactions Research Centre, School of Medical Sciences, Faculty of Medicine and Health, Örebro University, Örebro, Sweden

^2^Clinical Epidemiology and Biostatistics, School of Medical Sciences, Faculty of Medicine and Health, Örebro University, Örebro Sweden

^3^Wageningen Bioveterinary Research, Wageningen University and Research, Lelystad, the Netherlands

^4^BioGaia AB, Stockholm and Lund, Sweden

^5^Clinical Epidemiology Division, Department of Medicine, Solna, Karolinska Institutet, Stockholm, Sweden

^6^Department of Epidemiology and Public Health, University College London, London, UK

**Materials and methods**

***Severe acute respiratory syndrome coronavirus (SARS-CoV-2) virus neutralization test (VNT)***

Tissue culture medium: Minimum Essential Medium (MEM) with 5% Fetal Calf Serum (FCS), 1% Penicillin/Streptomycin, 1% Amphotericin, 1% L-glutamine, and 1% MEM Non-essential Amino Acids Solution

Test virus: SARS-CoV-2/Delta/IND_B.1.617.2/2021

Test cell: VeroE6-TMPRSS2 (NIBSC 100978), a cell line highly susceptible to SARS-CoV-2

Positive control serum 1: Anti-SARS-CoV-2 Ab NIBSC 20/136 (1:10 diluted)

Positive control serum 2: Bebtelovimab (1:500 diluted)

Negative control serum: Pooled pre-pandemic human sera

*VNT protocol:*

Heat-inactivated serum samples were serially diluted (first dilution 1:10 followed by a series of 3-fold dilutions) on 96-well plates and each sample dilution was incubated in duplicates for 1.5 h at room temperature in tissue culture medium containing ~100 TCID50 of SARS-CoV-2 (SARS-CoV-2/Delta/IND_B.1.617.2/2021). After incubation, VeroE6-TMPRSS2 (NIBSC 100978) cells were added to all wells at a concentration of 15000 cells/well. The plates were then incubated for 4-7 days at 37°C and 5% CO_2_ until a clear virus induced cytopathic effect (CPE) was visible. CPE was assessed via Immune Peroxidase Monolayer Assay (IPMA) staining which visualizes viral proteins. First, the cell monolayers were fixed with 4% formaldehyde, followed by a fixation with ice-cold 100% methanol. After the cells were washed with PBS, the cell monolayers were permeabilized with 1% Triton solution, washed with PBS-0.5% Tween-80 and subsequently blocked with PBS-5% horse serum solution. After blocking, the cell monolayers were treated with anti-spike (S1) antibody [Rabbit-anti-SARS-CoV-2-S1-2ST (619F) polyclonal serum against domain A of S1 (S1A, residues 1-294) protein, where the S1A is fused with triple Strep-tag and produced in HEK293T cells; the rabbit serum was prepared by Davids Biotechnologie GmbH]. Then the monolayers were washed with PBS-Tween 80 solution after which they were treated with goat-anti-rabbit-HRP (Dako), washed, incubated with 3-amino-9-ethylcarbazole substrate solution until a red-brown color developed.

*Interpretation of the results:*

All 96-well plates were read-out independently by two trained technicians according to the 4-eye principle. The technicians were blinded to the group identities.

The neutralization titer of each sample was determined as the reciprocal dilution at which 50% or less of the cell monolayer is stained for virus antigen (i.e., virus neutralization). The 50% determination was done visually. Each sample was analyzed in duplicate and the log10 of the titres of the duplicates were averaged to obtain the virus neutralization titer for each sample.

The detection limit for the assay in this experimental setting was a titer of 5.77. Thus, a value <6 was assigned to all samples with an undetectable titer.

*Test validation:*

The VNT is considered valid when the virus dose per well, as determined by the back titration, is between 15 and 300 virus particles per well. The virus used in the test was back titrated in a two-fold dilution series and each dilution was tested in 8 wells. The virus back titration titer found in this VNT run, determined by the Spearman-Kärber algorithm was 107 TCID50/well.

The negative control serum showed no virus neutralization capacity (titer <6) whereas the positive controls Anti-SARS-CoV-2 Ab NIBSC 20/136 and bebtelovimab showed neutralization titers of 173 and 135000, respectively.

**Supplementary Table 1.** Participant characteristics, anti-SARS-CoV-2-specific antibody titers, and COVID-19 associated symptoms in SARS-CoV-2-infected individuals

|  | | |  | ITT population | | | PP population | | |
| --- | --- | --- | --- | --- | --- | --- | --- | --- | --- |
| Group | | |  | Placebo | Probiotic | p | Placebo | Probiotic | p |
| n | | |  | 7 | 8 |  | 6 | 7 |  |
| Age (years) | | | | 48 (39-60) | 49.5 (41-58) |  | 51 (39-60) | 48 (41-58) |  |
| Sex | |  | |  |  |  |  |  |  |
|  | Female | | | 5 | 5 |  | 5 | 5 |  |
|  | Male | | | 2 | 3 |  | 1 | 2 |  |
| Antibody (BAU/ml) | | | |  |  |  |  |  |  |
|  | Anti-S IgG | | | 111 (36.1-1210)* | 609 (168-1480)* | 0.080 | 92.7 (36.1-1210)# | 579 (168-755)# | 0.654 |
|  | Anti-RBD IgG | | | 83.7 (22.8-2094)* | 928 (212-3449)* | 0.066 | 93 (22.8-2094)# | 751 (212-3449)# | 0.188 |
|  | Anti-N IgG | | | 158 (15.1-2086) | 331 (25.2-1784) | 0.253 | 147 (15.1-2086) | 226 (25.2-1784) | 0.414 |
|  | Anti-S IgA | | | 1123 (127-6711)* | 763 (254-2521)* | 0.977 | 214 (127-6711)# | 619 (254-920)# | 0.979 |
|  | Anti-RBD IgA | | | 252 (81.4-6714)* | 708 (210-1590)* | 0.595 | 187 (81.4-2605)# | 838 (210-1590)# | 0.240 |
|  | Anti-N IgA | | | 764 (61.2-38339) | 687 (24.1-1343) | 0.444 | 579 (61.2-1202) | 426 (24.1-1343) | 0.991 |
| SARS-CoV-2 nAB titer | | | | 30 (10-90)* | 41 (30-90)* | 0.546 | 30 (10-52)# | 30 (30-90)# | 0.484 |
| Days from infection to sampling | | | | 28 (9-97) | 33.5 (7-56) | 0.977 | 39 (9-97) | 31 (7-56) | 0.865 |
| Symptom duration (days) | | | | 14 (12-26) | 17 (6-31) | 0.843 | 16.5 (13-26) | 23 (6-31) | 0.916 |
| Symptom severity | | | |  |  |  |  |  |  |
|  | Ambulatory | | | 7 | 8 |  | 6 | 7 |  |
|  | Mild | | | - | - |  | - | - |  |
|  | Severe | | | - | - |  | - | - |  |
| Frequency of symptoms | | | |  |  |  |  |  |  |
|  | Cough | | | 4 | 7 |  | 4 | 7 |  |
|  | Sore throat | | | 1 | 2 |  | 1 | 1 |  |
|  | Nasal congestion | | | 3 | 6 |  | 1 | 6 |  |
|  | Breathing difficulties | | | 1 | 1 |  | 1 | 1 |  |
|  | Fever | | | 4 | 7 |  | 3 | 6 |  |
|  | Body aches | | | 3 | 5 |  | 2 | 5 |  |
|  | Loss of taste or smell | | | 3 | 3 |  | 2 | 3 |  |
|  | Diarrhea | | | - | 1 |  | - | 1 |  |
|  | Nausea | | | - | - |  | - | - |  |
|  | Headache | | | 4 | 7 |  | 4 | 6 |  |
|  | Fatigue | | | 7 | 8 |  | 6 | 7 |  |
| Total daily energy intake (mJ) | | | | 7.7 (6.5-11.7) | 7.3 (4.8-9.4) | 0.335 | 8.1 (6.5-11.7) | 7.0 (4.8-9.4) | 0.181 |
| Daily intake (% of total daily energy intake) | | | |  |  |  |  |  |  |
|  | Carbohydrates | | | 44.3 (33.8-56.8) | 42.2 (32.7-50.4) | 0.779 | 40.3 (33.8-49.7) | 40.2 (32.7-50.4) | 0.945 |
|  | Protein | | | 16.6 (11.0-20.7) | 17.6 (14.0-20.9) | 0.672 | 17.1 (15.1-20.7) | 18.2 (14-20.9) | 0.915 |
|  | Fat | | | 35.3 (26.6-45.3) | 35.0 (30.2-43.2) | 0.955 | 37.3 (30.1-45.3) | 35.0 (30.2-43.2) | 0.836 |
|  | Fiber | | | 4.3 (1.9-5.1) | 2.3 (1.5-3.8) | 0.06 | 3.5 (1.9-5.1) | 2.4 (1.5-3.8) | 0.149 |
|  | Alcohol | | | 1.3 (0-2.8) | 2.9 (1.1-5.6) | 0.02 | 1.2 (0-2.8) | 2.9 (1.1-5.6) | 0.06 |

*n=6, #n=5, S = spike, RBD = receptor-binding domain, N = nucleocapsin, nAB = neutralizing antibody, ITT = intention to treat, PP = per protocol

**Supplementary Table 2.** Participant characteristics, anti-SARS-CoV-2-specific antibody titers, and COVID-19 vaccine information in fully vaccinated individuals

|  | |  | ITT population | | | PP population | | | | |
| --- | --- | --- | --- | --- | --- | --- | --- | --- | --- | --- |
| Group | |  | Placebo | Probiotic | p | Placebo | Probiotic | | p | |
| n | |  | 19 | 25 |  | 16 | 23 | |  | |
| Age (years) | | | 51 (31-60) | 51 (21-60) |  | 51.5 (31-60) | | 52 (21-60) | |  |
| Sex | | |  |  |  |  |  | |  | |
|  | Female | | 15 | 23 |  | 14 | 22 | |  | |
|  | Male | | 4 | 2 |  | 2 | 1 | |  | |
| Antibody (BAU/ml) | | |  |  |  |  |  | |  | |
|  | Anti-S IgG | | 1340 (253-11000) | 1680 (756-14000) | 0.221 | 1325 (253-11000) | 1680 (756-14000) | | 0.301 | |
|  | Anti-RBD IgG | | 2068 (387-20341) | 3215 (1321-16258) | 0.127 | 1978 (387-20341) | 2985 (1321-16258) | | 0.203 | |
|  | Anti-S IgA | | 147 (21.9-2617) | 297 (43.9-5996) | 0.266 | 317 (21.9-2617) | 273 (43.9-5996) | | 0.732 | |
|  | Anti-RBD IgA | | 69.1 (20.5-1771) | 186 (32.9-5940) | 0.118 | 147 (20.5-1771) | 183 (32.9-5940) | | 0.419 | |
| SARS-CoV-2 nAB titer | | | 52 (17-468) | 90 (30-468) | 0.996 | 71 (17-468) | 90 (30-468) | | 0.676 | |
| Days from 2^nd^ vaccine dose to sampling | | | 27 (8-112) | 27 (5-71) | 0.529 | 25.5 (8-78) | 27 (5-71) | | 0.949 | |
| Days between vaccine doses | | | 42 (23-70) | 42 (22-72) |  | 42.5 (23-70) | 42 (22-72) | |  | |
| Vaccine types | | |  |  |  |  |  | |  | |
|  | BNT162b2 | | 16 | 22 |  | 13 | 20 | |  | |
|  | mRNA-1273 | | - | 2 |  | - | 2 | |  | |
|  | AZD1222 | | 3 | - |  | 3 | - | |  | |
|  | AZD1222 + BNT162b2 | | - | 1 |  | - | 1 | |  | |
| Total daily energy intake (mJ) | | | 7.7 (4.7-11.8) | 9.3 (4.9-14.2) | 0.036 | 7.7 (4.7-10.3) | 9.3 (4.9-14.2) | | 0.018 | |
| Dietary intake (% of total daily energy intake) | | |  |  |  |  |  | |  | |
|  | Carbohydrates | | 42.4 (20.6-49.2) | 42.4 (25.5-52.5) | 0.578 | 42.4 (20.6-49.2) | 42.4 (29.5-52.5) | | 0.536 | |
|  | Protein | | 16.9 (13.5-20.9) | 16.4 (11.1-19.5) | 0.376 | 16.9 (13.5-20.9) | 16.2 (11.1-19.5) | | 0.246 | |
|  | Fat | | 36.1 (28.2-55.4) | 36.9 (30.2-51.0) | 0.614 | 36.1 (28.2-55.4) | 36.9 (30.2-47.2) | | 0.641 | |
|  | Fiber | | 2.7 (1.9-3.1) | 2.7 (1.4-4.1) | 0.403 | 2.6 (1.9-3.1) | 2.8 (1.4-4.1) | | 0.505 | |
|  | Alcohol | | 1.7 (0.1-10.3) | 1.0 (0-7.7) | 0.491 | 1.7 (0.1-10.3) | 1.0 (0-7.7) | | 0.140 | |

S = spike, RBD = receptor-binding domain, nAB = neutralizing antibody, ITT = intention to treat, PP = per protocol

**Supplementary figures**

**Supplementary Figure 1.** Correlations between serum levels of different anti-SARS-CoV-2-specific antibodies and the number of days from the second COVID-19 vaccine dose. S = spike, RBD = receptor-binding domain, nAB = neutralizing antibody. Orange dots represent individuals in the active treatment arm, black dots individuals in the placebo group.

**Supplementary Figure 2.** Correlations between serum levels of different anti-SARS-CoV-2-specific antibodies and virus-neutralizing antibody titers. S = spike, RBD = receptor-binding domain, nAB = neutralizing antibody. Orange dots represent individuals in the active treatment arm, black dots individuals in the placebo group.
